# Supplementary material for: An antifungal polyketide associated with horizontally acquired genes supports symbiont-mediated defense in Lagria villosa beetles
Source: Nat Commun. 2018 Jun 26;9:2478. doi: 10.1038/s41467-018-04955-6 (PMC6018673; doi:10.1038/s41467-018-04955-6)
Supplement: Supplementary file 1 — Supplementary Information [file 41467_2018_4955_MOESM1_ESM.pdf]

Supplementary Information

An antifungal polyketide associated with horizontally acquired genes supports symbiont-mediated defense in *Lagria villosa* beetles

Laura V. Flórez, Kirstin Scherlach, Ian J. Miller, Andre Rodrigues, Jason C. Kwan, Christian Hertweck and Martin Kaltenpoth

Supplementary Note 1. Physicochemical properties of lagriamide

ESI(+) *m/z* 749 $(M+H)^+$, HRESI(+)-MS *m/z* 749.4949 (calcd. for $C_{41}H_{69}N_2O_{10}$ 749.4947)

UV (PDA): λ_{max}=UVend

NMR: d_6-DMSO, ^1H NMR 600 MHz, ^{13}C NMR 150 MHz

Carbon	^{13}C	^1H (mult., *J* in Hz)
**1**	17.2	1.29 (d, *5.1*)
**2**	53.7	3.10 (m)
**3**	59.7	3.34 (d, *2.0*)
**4**	205.4	-
**5**	44.6	2.47 (d, *6.4*)
**6**	74.1	3.70 (m)
**7**	25.4	1.33 (m)
**8**	30.2	1.71 (m)<br>1.59 (m)
**9**	29.3	1.60 (m)
**10**	11.6	0.85 (d, *7.0*)
**11**	76.6	3.77 (m)
**12**	39.8	2.20 (dd, *14.5, 8.3*)<br>2.06 (dd, *14.4, 5.4*)
**13**	170.2	-
**14**	42.9	3.21 (m)<br>2.92 (dt, *13.0*, *5.8*)
**15**	71.4	3.50 (m)
**16**	43.5	2.25 (m)
**17**	14.4	0.95 (d, *7.0*)
**18**	174.1	-
**19**	38.6	3.05 (m)
**20**	25.6	1.69 (m)<br>1.35 (m)
**21**	30.1	1.53 (m)<br>1.20 (m)
**22**	73.8	3.06 (m)
**23**	34.4	1.20 (m)
**24**	17.9	0.76 (d, *6.5*)
**25**	27.6	1.41 (m)
**26**	35.5	1.50 (m)<br>1.34 (m)
**27**	94.6	-

**28**	34.9	1.44 (m)
**29**	18.8	1.49 (m)
**30**	31.0	1.47 (m)<br>1.05 (m)
**31**	68.0	3.40 (m)
**32**	33.4	1.41 (m)*<br>1,27 (m)
**33**	33.3	1.27 (m)*
**34**	31.4	2.30 (m)
**35**	21.2	0.86 (d, *6.6*)
**36**	131.0	4.87 (d, *9.1*)
**37**	132.4	-
**38**	16.0	1.55 (d, *1.2*)
**39**	34.4	2.15 (m)
**40**	33.0	2.24 (m)
**41**	174.3	-

* overlapping signals, might be interchangeable

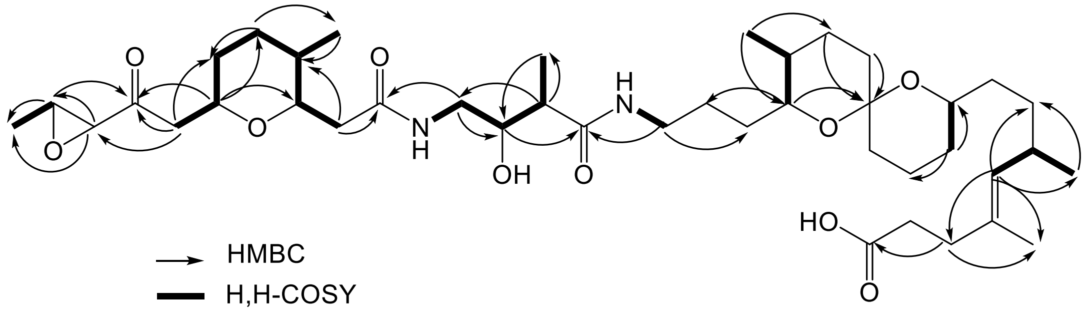

Supplementary Figure 1. Key 2D NMR couplings of lagriamide.

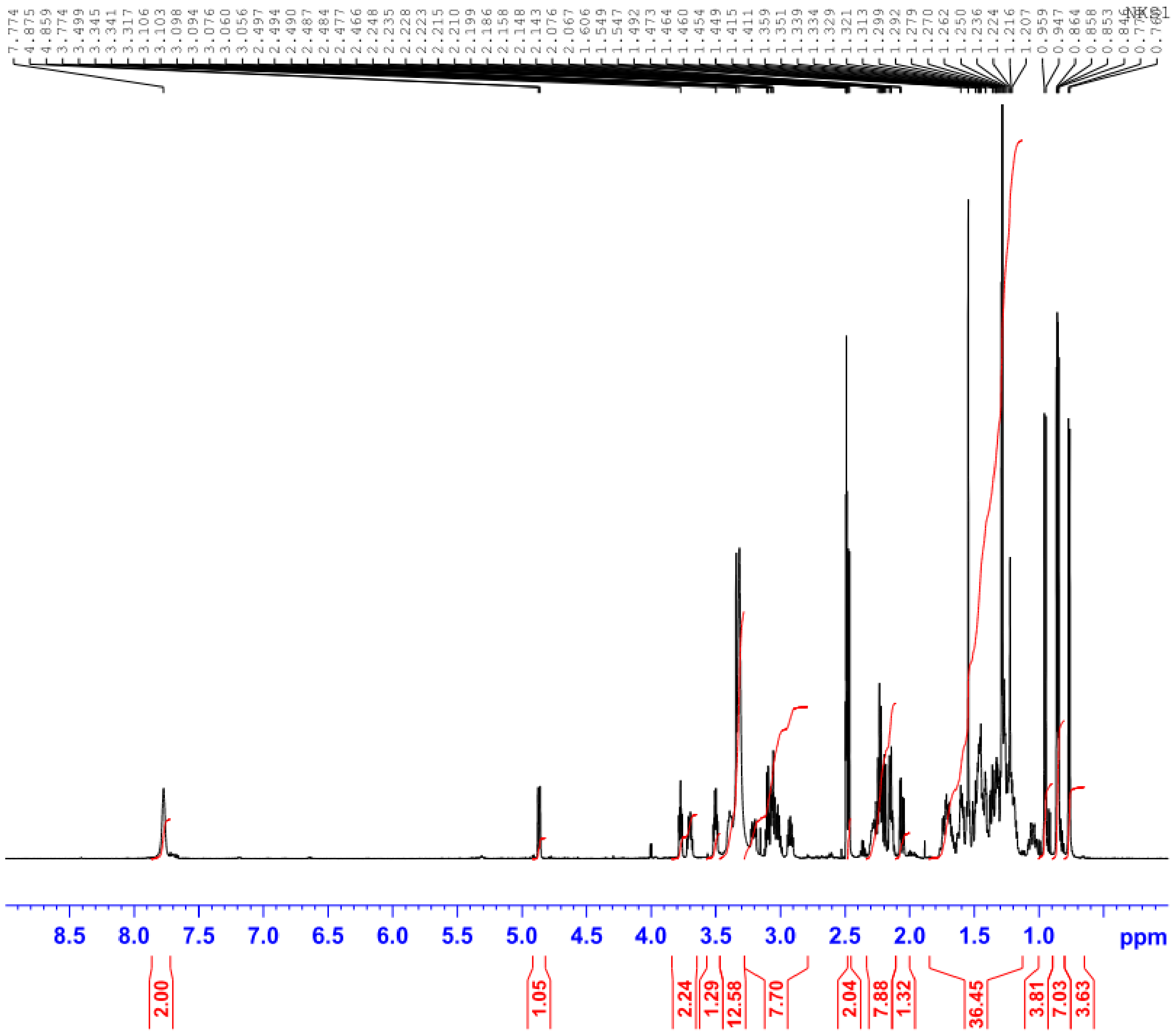

Supplementary Figure 2. ¹H NMR spectrum of lagriamide.

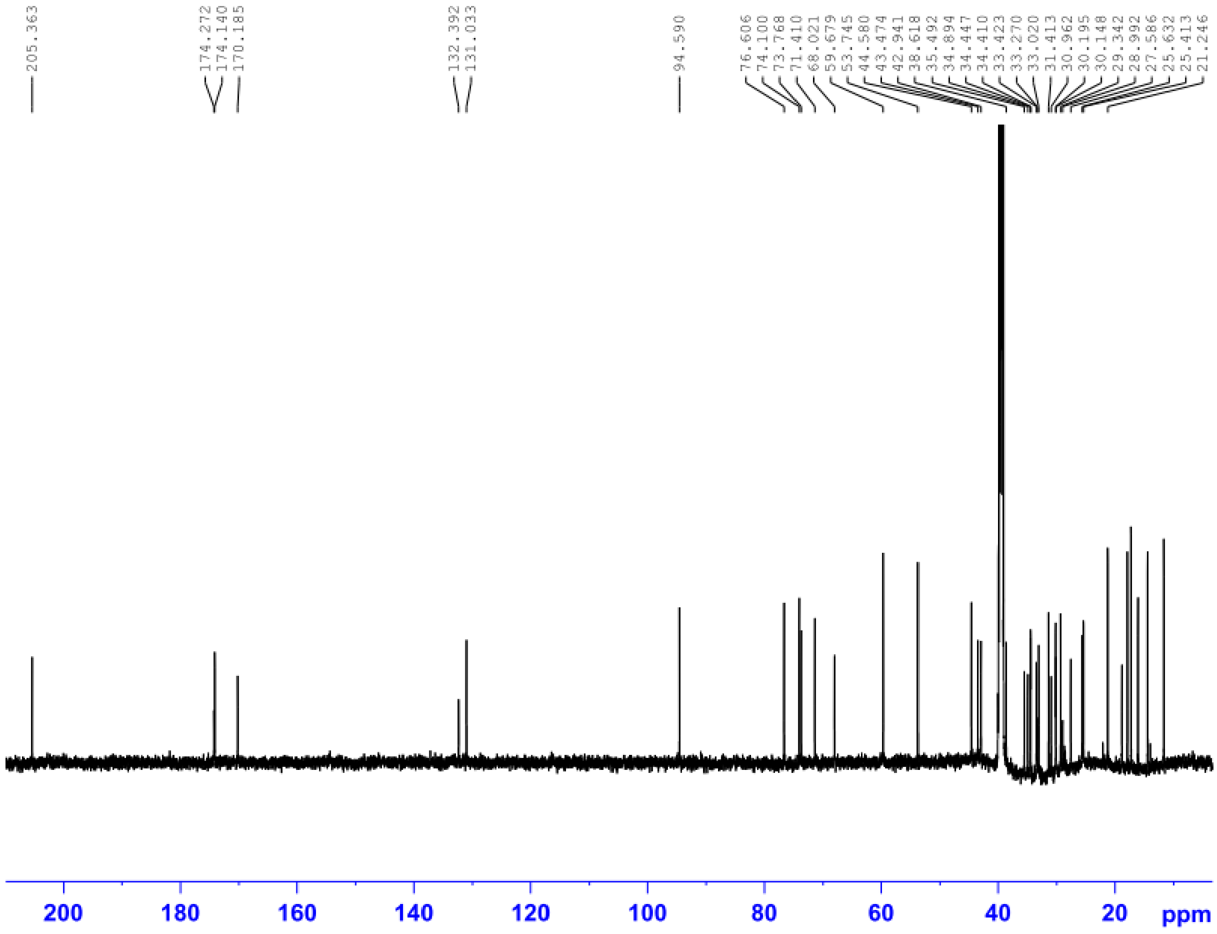

Supplementary Figure 3. ^{13}C NMR spectrum of lagriamide.

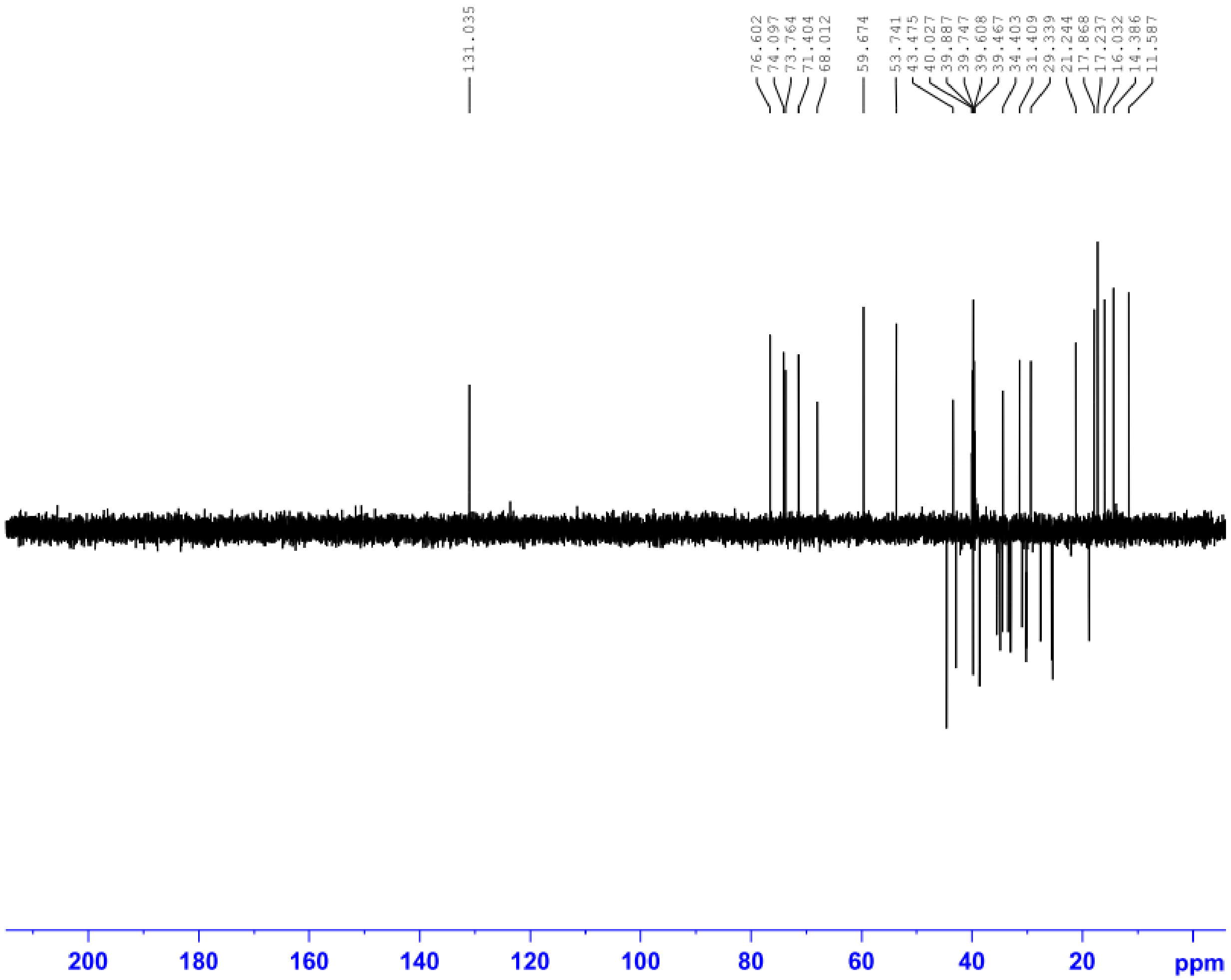

Supplementary Figure 4. DEPT135 NMR spectrum of lagriamide.

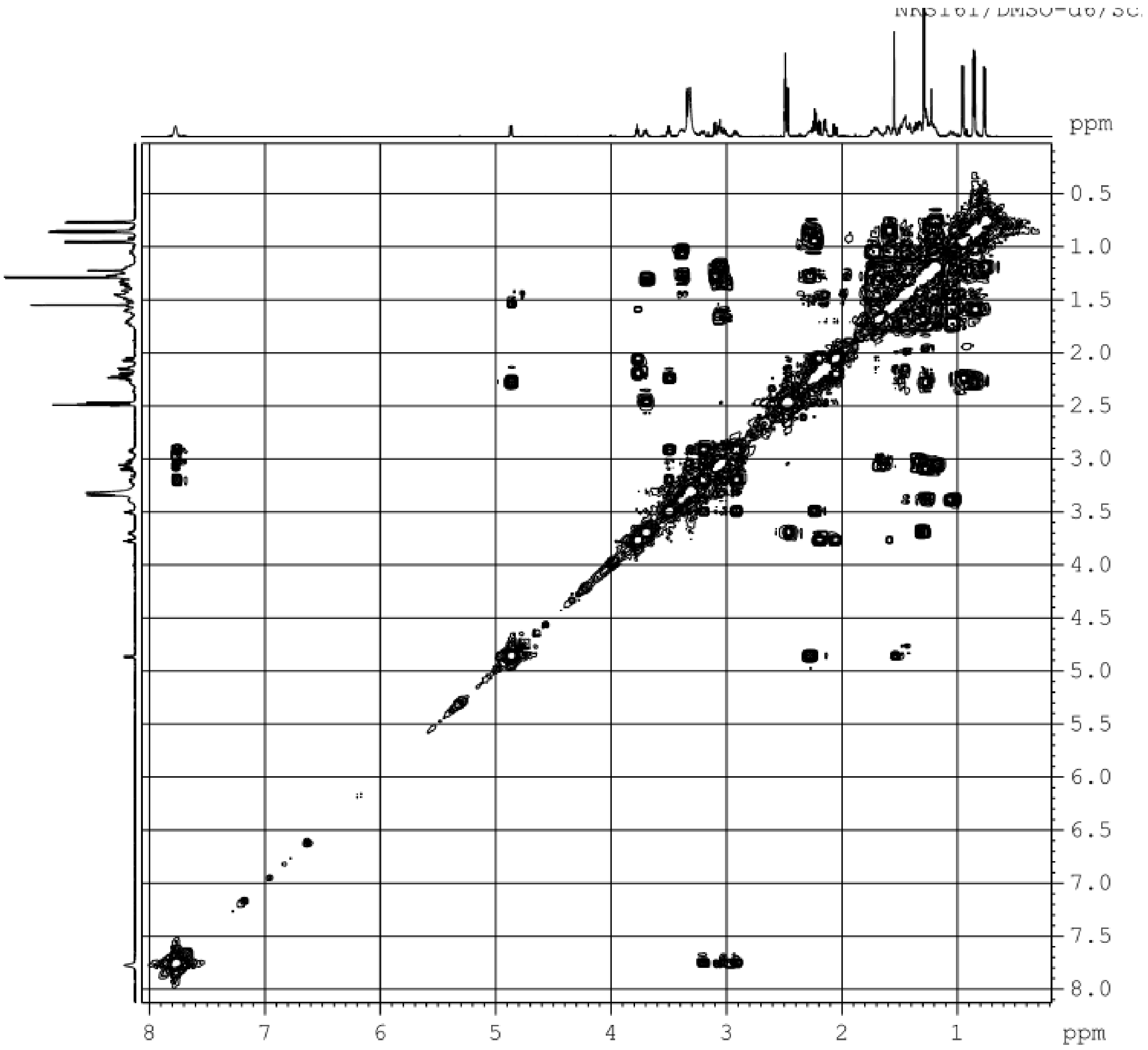

Supplementary Figure 5. H,H-COSY spectrum of lagriamide.

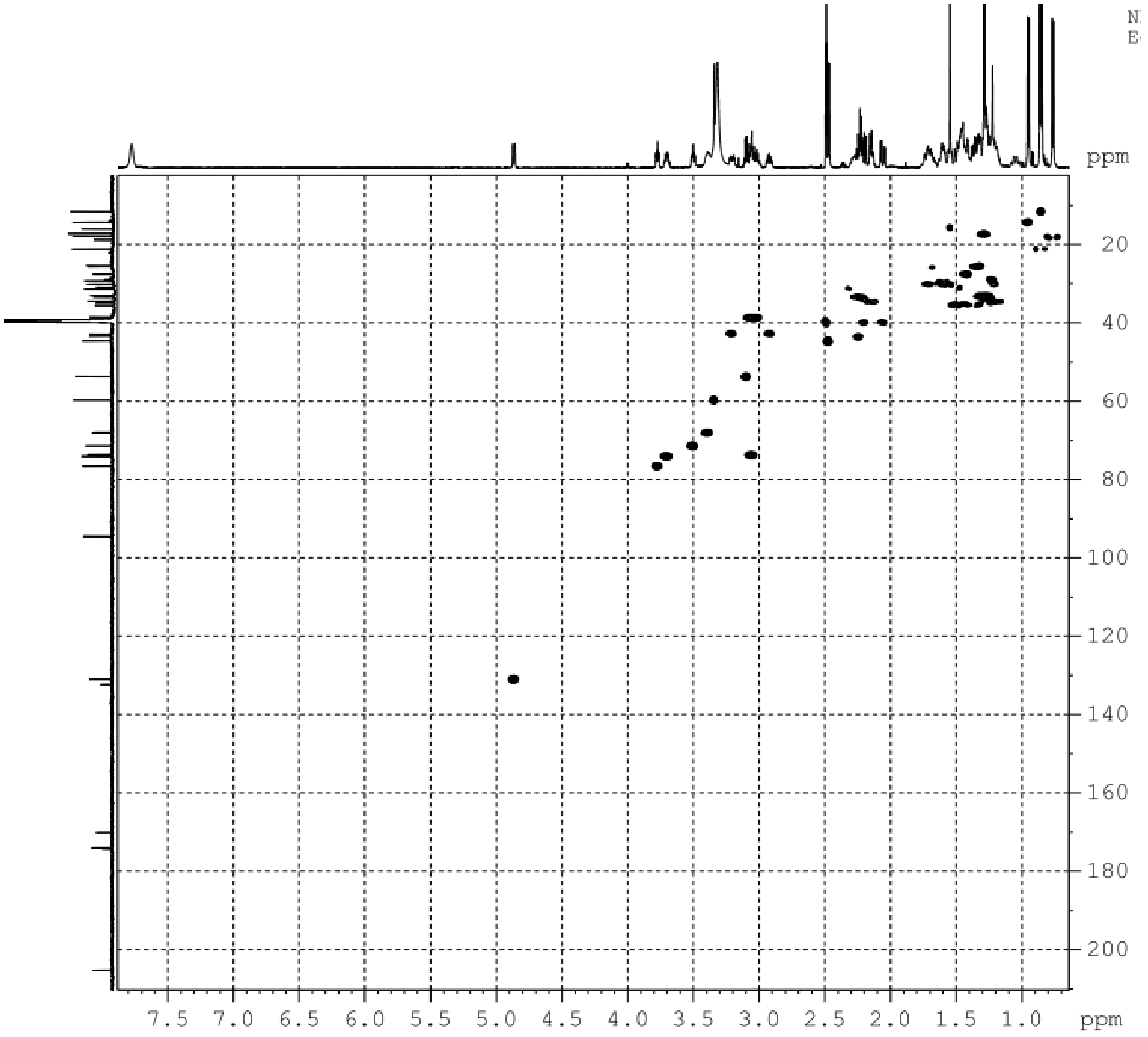

Supplementary Figure 6. HSQC spectrum of lagriamide.

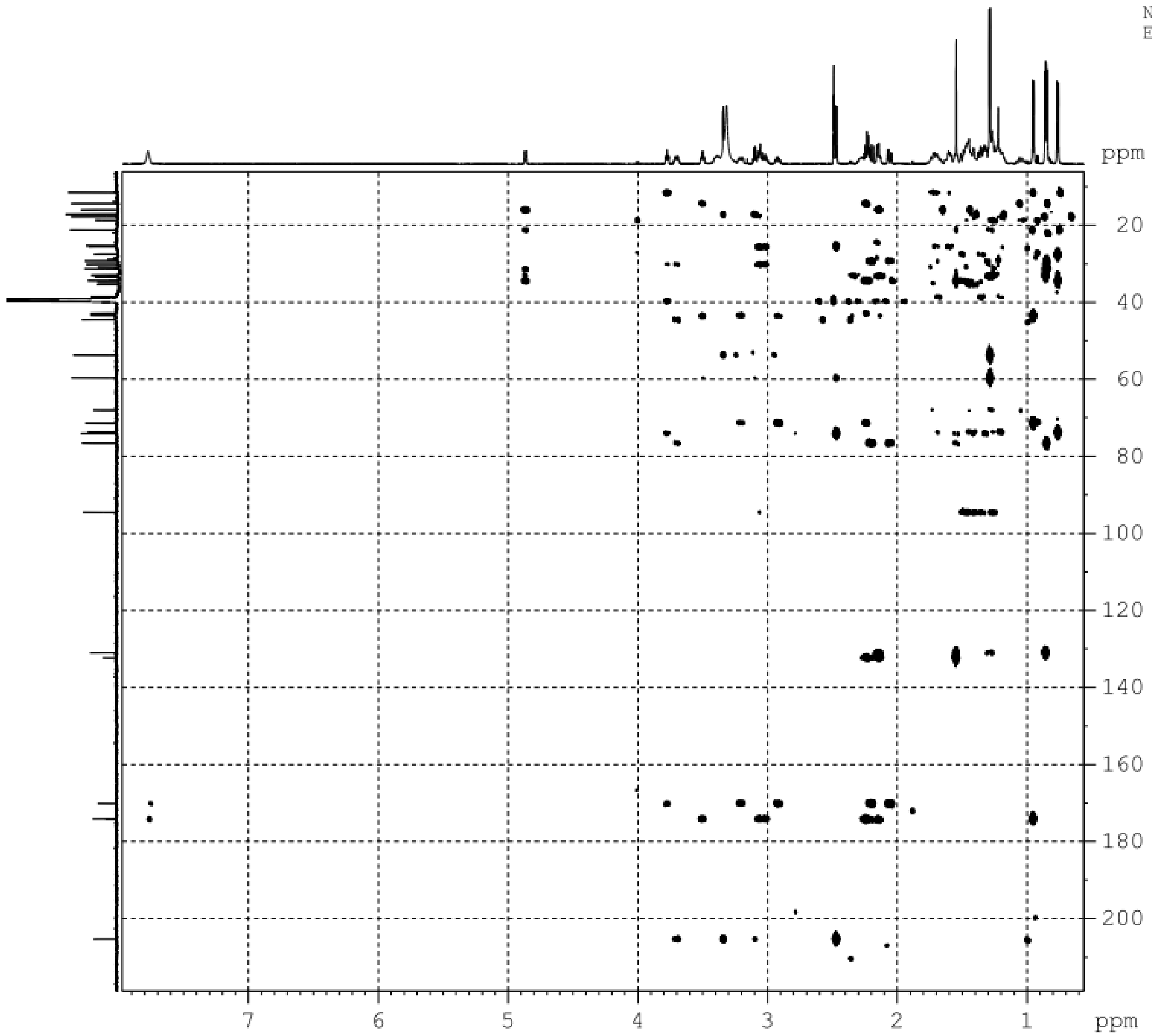

Supplementary Figure 7. HMBC spectrum of lagriamide.

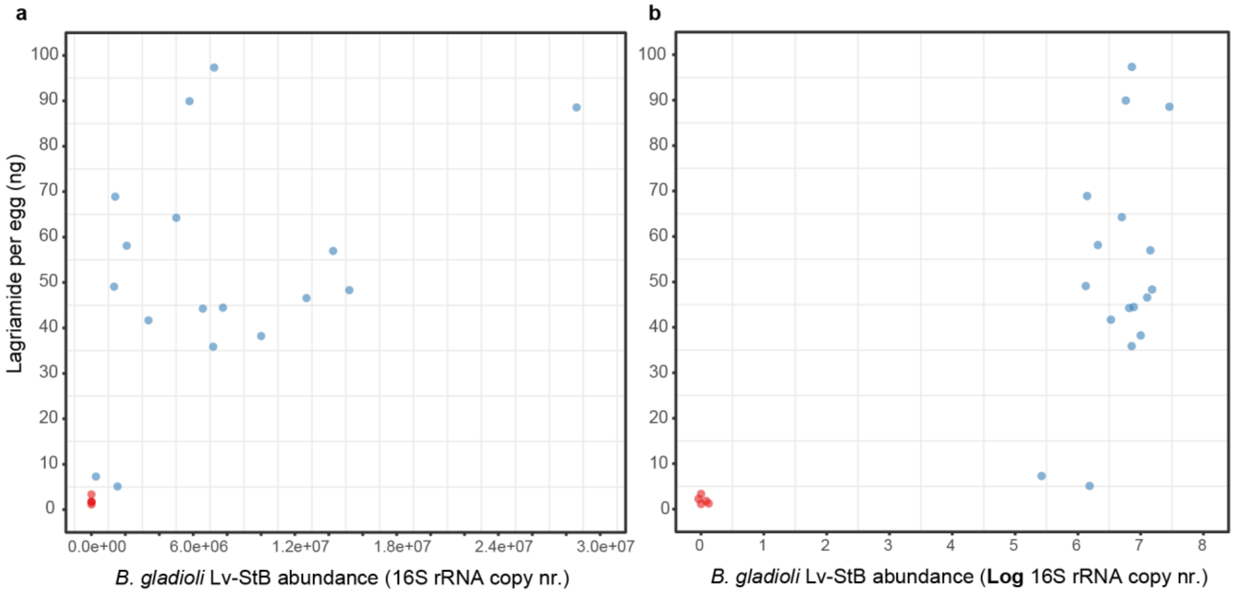

Supplementary Figure 8. Lagriamide amount per egg determined using 22 independent clutches laid by field-collected *Lagria villosa* and the corresponding titers of *Burkholderia gladioli* Lv-StB as assessed by qPCR of the 16S rRNA of this specific strain (Spearman Rank correlation, $p < 0.01$, rho = 0.625, N = 22). Symbiont titers are shown as (**a**) untransformed copy number and (**b**) log transformed copy number. Circle color indicates clutches in which *B. gladioli* Lv-StB was detected (blue) or not (red).

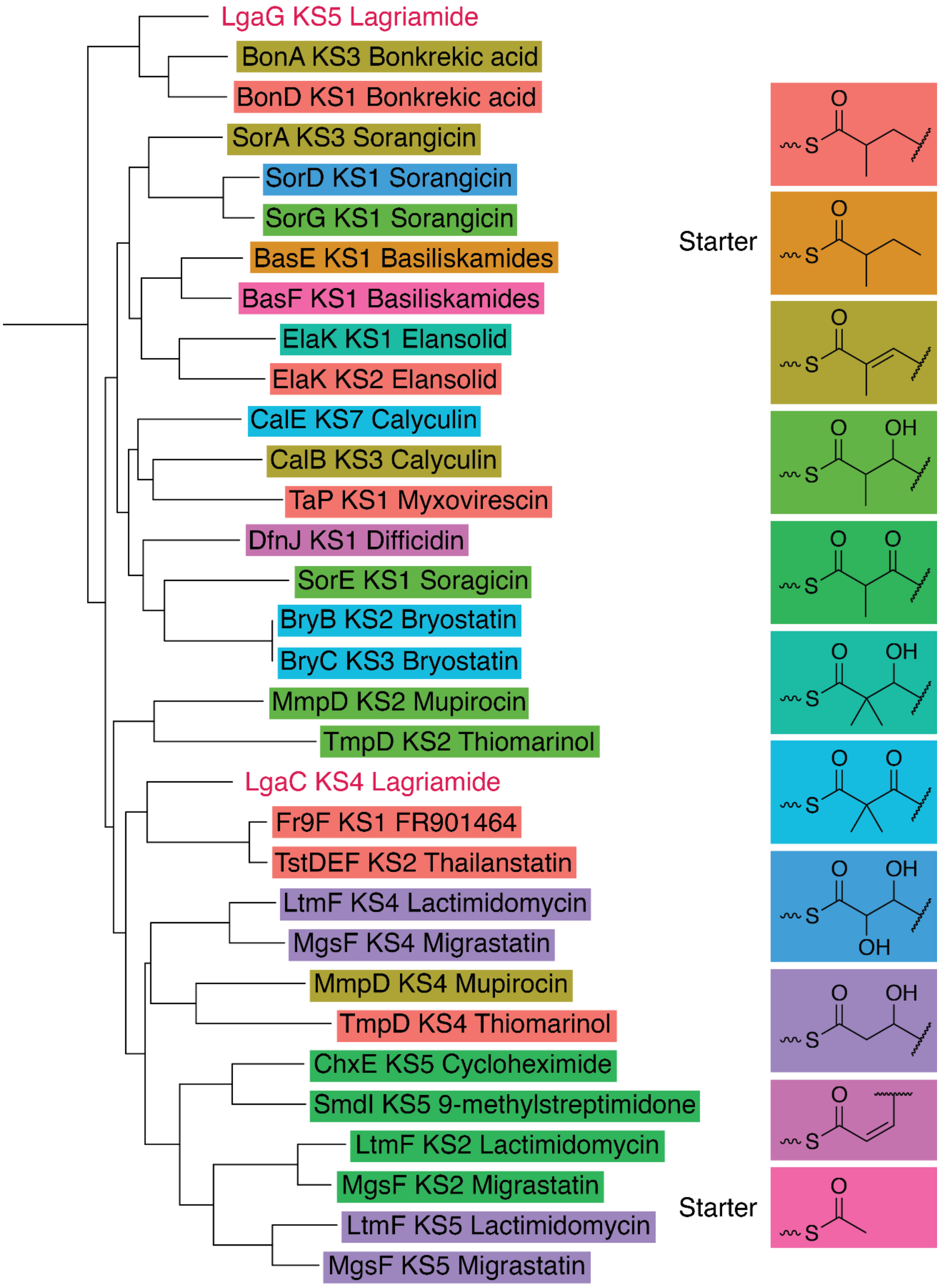

Supplementary Figure 9. Expansion of a clade containing *lga* KS domains in an approximately maximum-likelihood tree made from 666 *trans*-AT KS domain sequences, with KS domains from the erythromycin pathway acting as an outgroup. Colors correspond to the substrate chemical structures shown to the right of the clade.

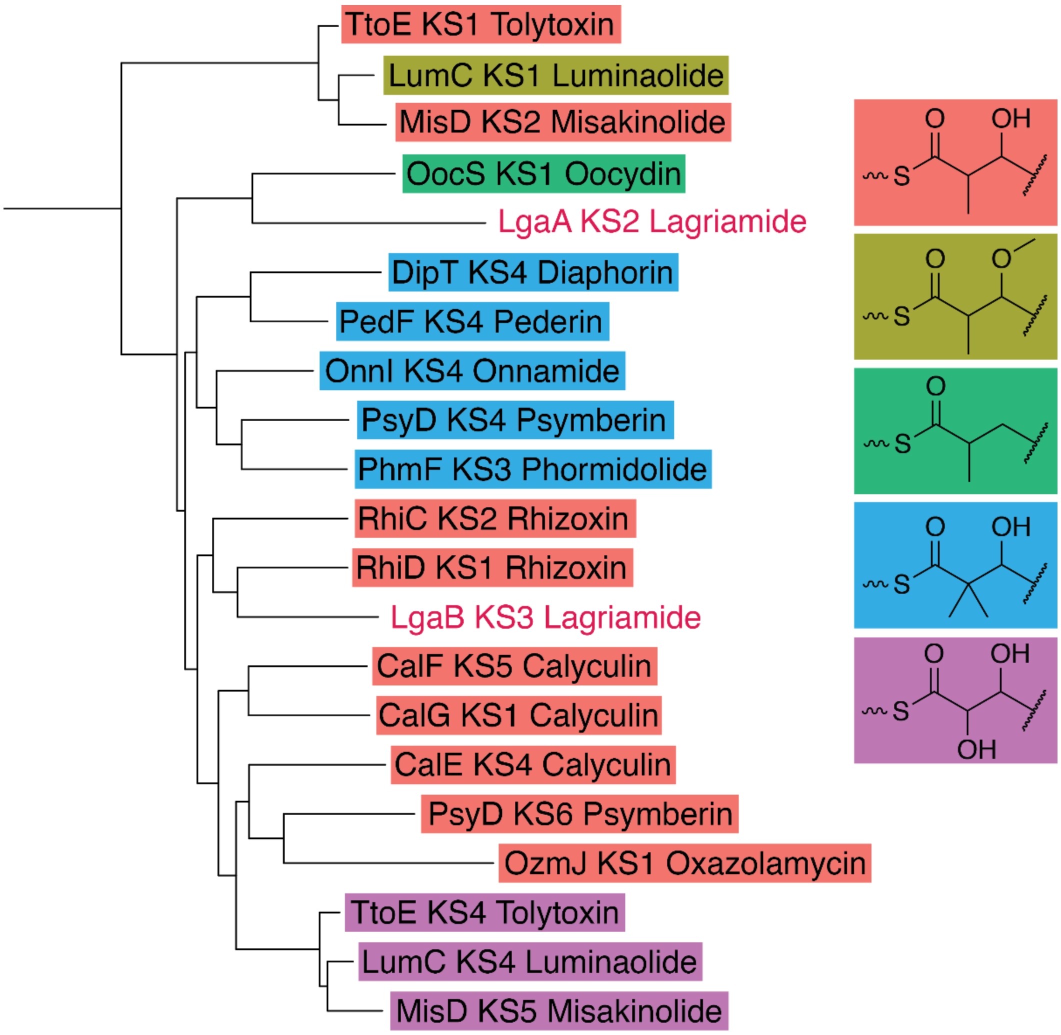

Supplementary Figure 10. Expansion of a clade containing *lga* KS domains in an approximately maximum-likelihood tree made from 666 *trans*-AT KS domain sequences, with KS domains from the erythromycin pathway acting as an outgroup. Colors correspond to the substrate chemical structures shown to the right of the clade.

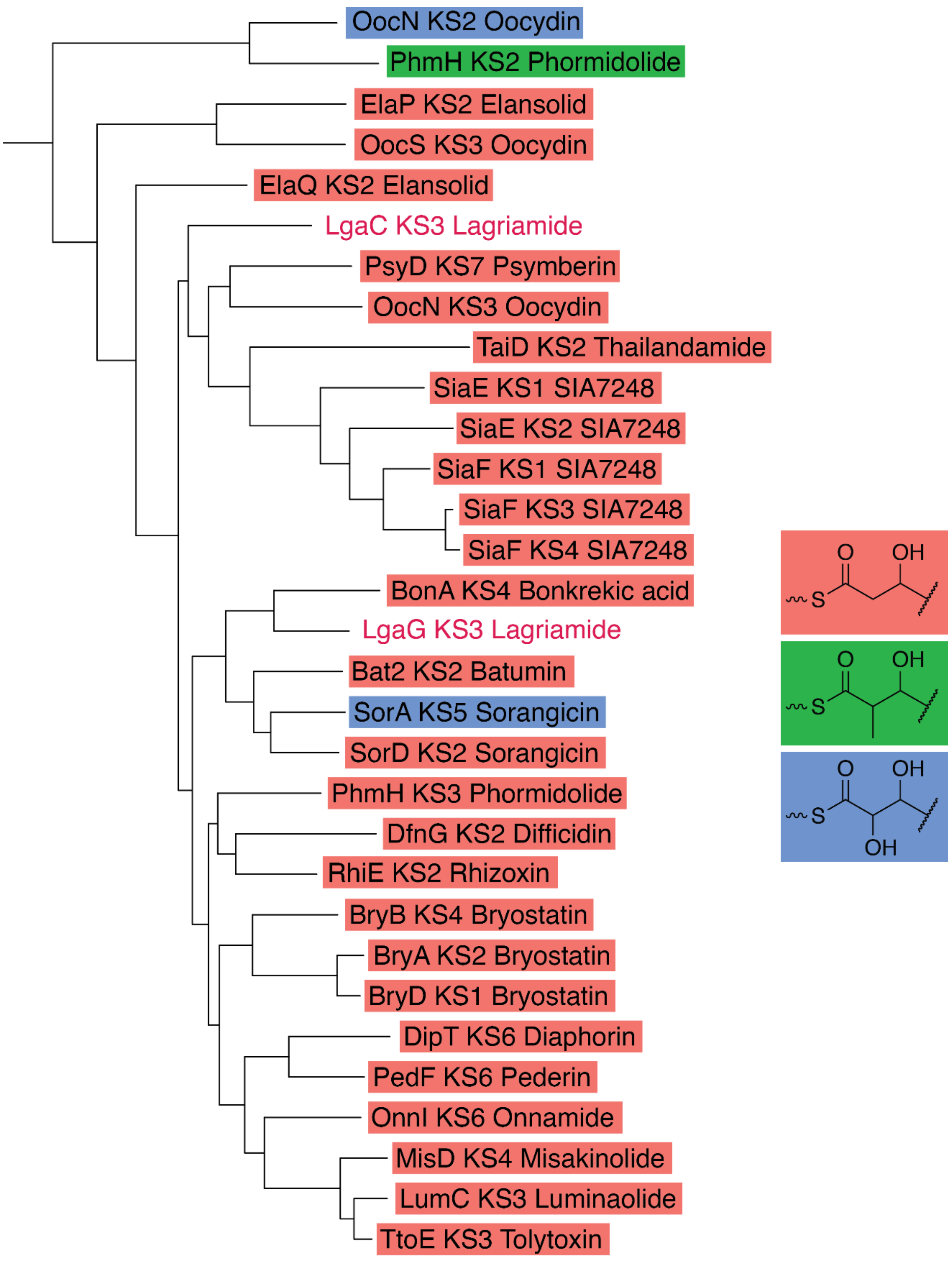

Supplementary Figure 11. Expansion of a clade containing *lga* KS domains in an approximately maximum-likelihood tree made from 666 *trans*-AT KS domain sequences, with KS domains from the erythromycin pathway acting as an outgroup. Colors correspond to the substrate chemical structures shown to the right of the clade.

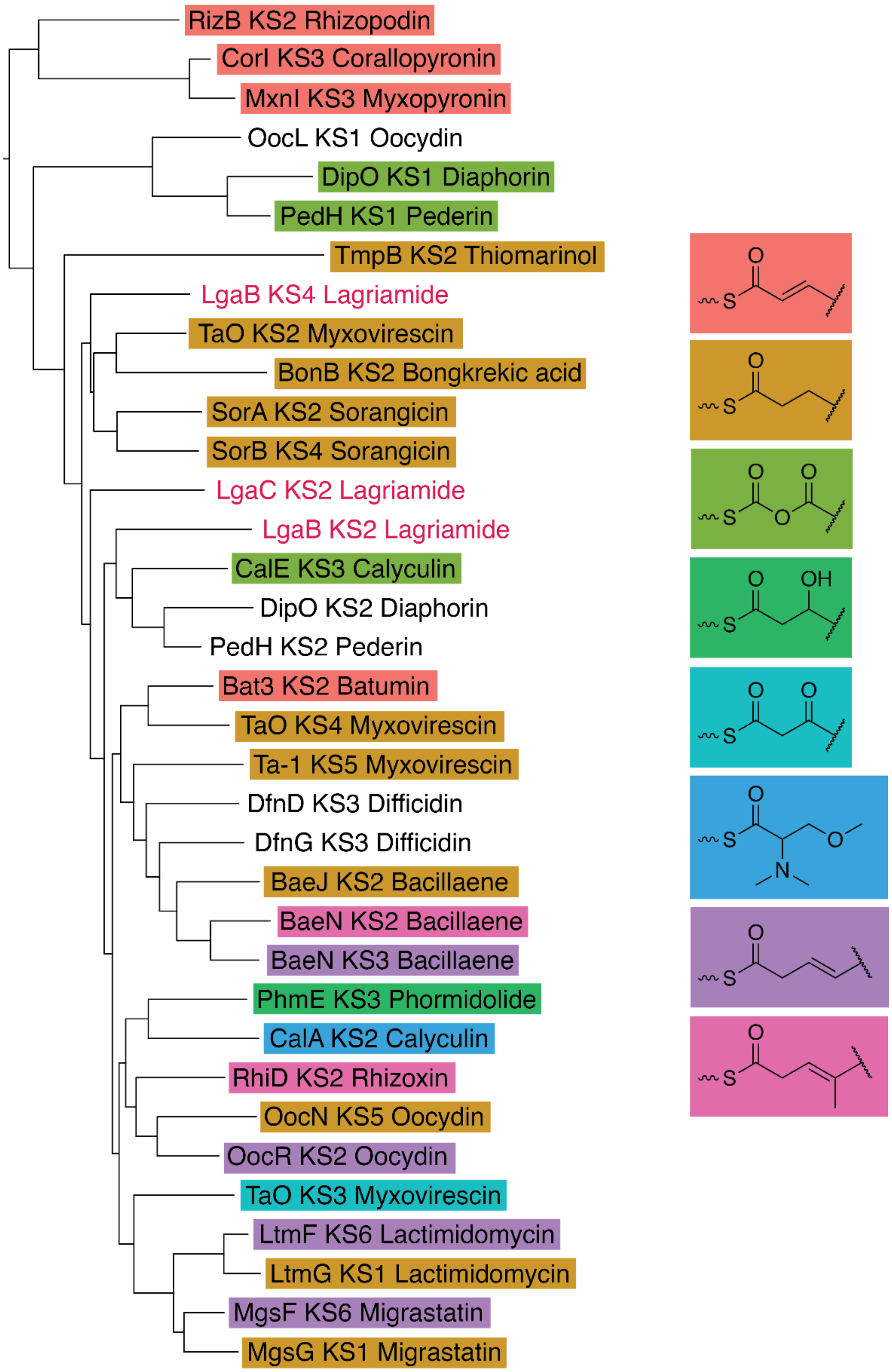

Supplementary Figure 12. Expansion of a clade containing *lga* KS domains in an approximately maximum-likelihood tree made from 666 *trans*-AT KS domain sequences, with KS domains from the erythromycin pathway acting as an outgroup. Colors correspond to the substrate chemical structures shown to the right of the clade.

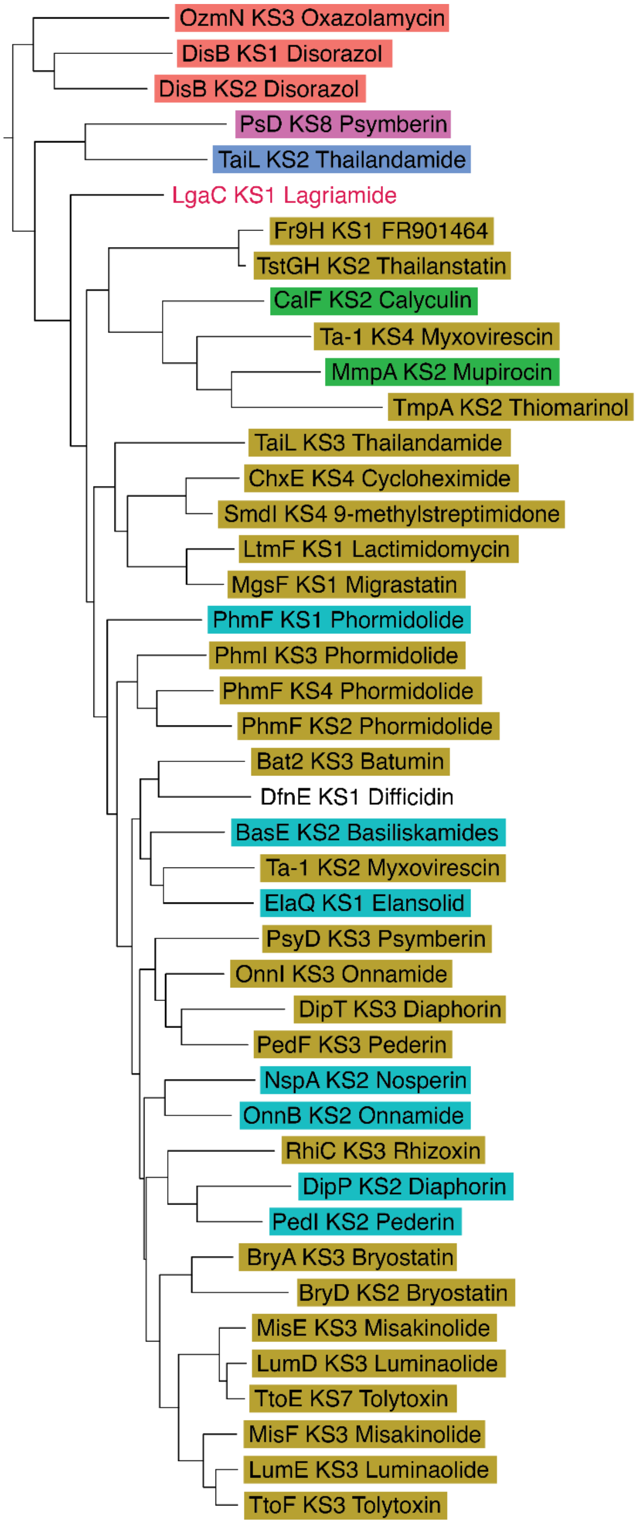

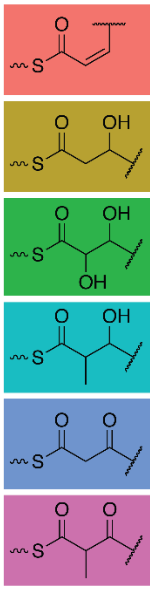

Supplementary Figure 13. Expansion of a clade containing *lga* KS domains in an approximately maximum-likelihood tree made from 666 *trans*-AT KS domain sequences, with KS domains from the erythromycin pathway acting as an outgroup. Colors correspond to the substrate chemical structures shown to the right of the clade.

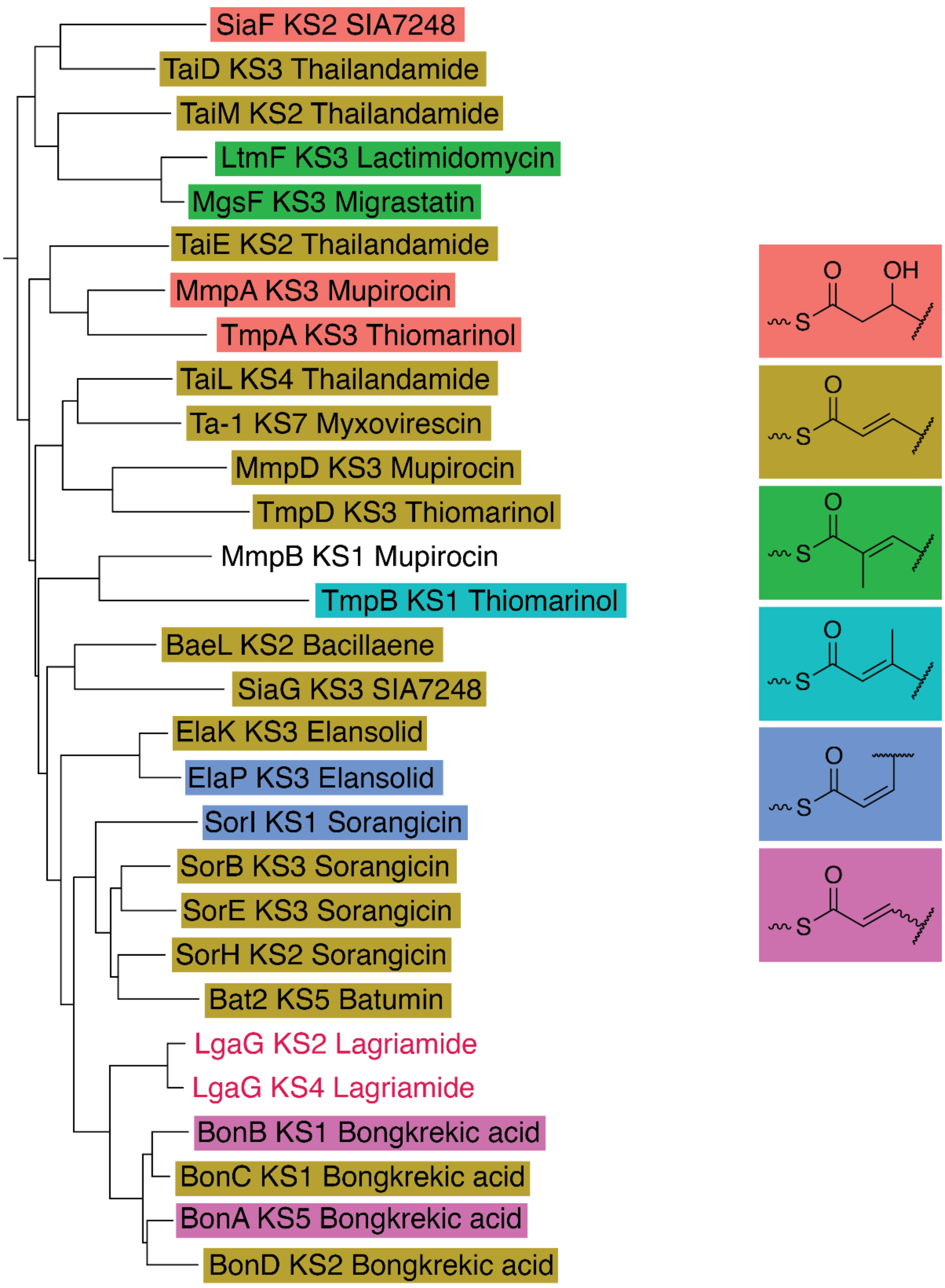

Supplementary Figure 14. Expansion of a clade containing *lga* KS domains in an approximately maximum-likelihood tree made from 666 *trans*-AT KS domain sequences, with KS domains from the erythromycin pathway acting as an outgroup. Colors correspond to the substrate chemical structures shown to the right of the clade.

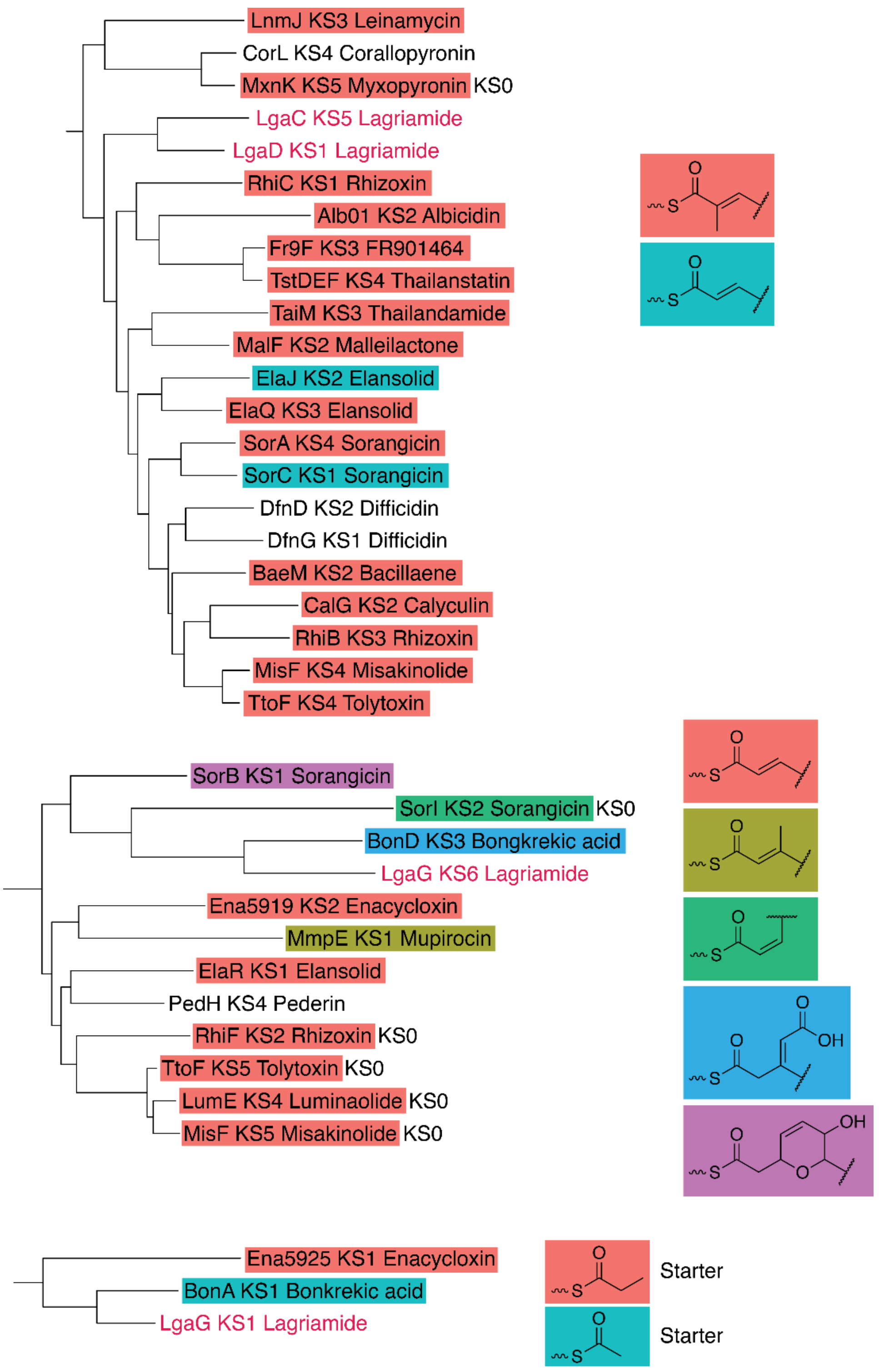

Supplementary Figure 15. Expansion of three clades containing *lga* KS domains in an approximately maximum-likelihood tree made from 666 *trans*-AT KS domain sequences, with KS domains from the erythromycin pathway acting as an outgroup. Colors correspond to the substrate chemical structures shown to the right of the clade.

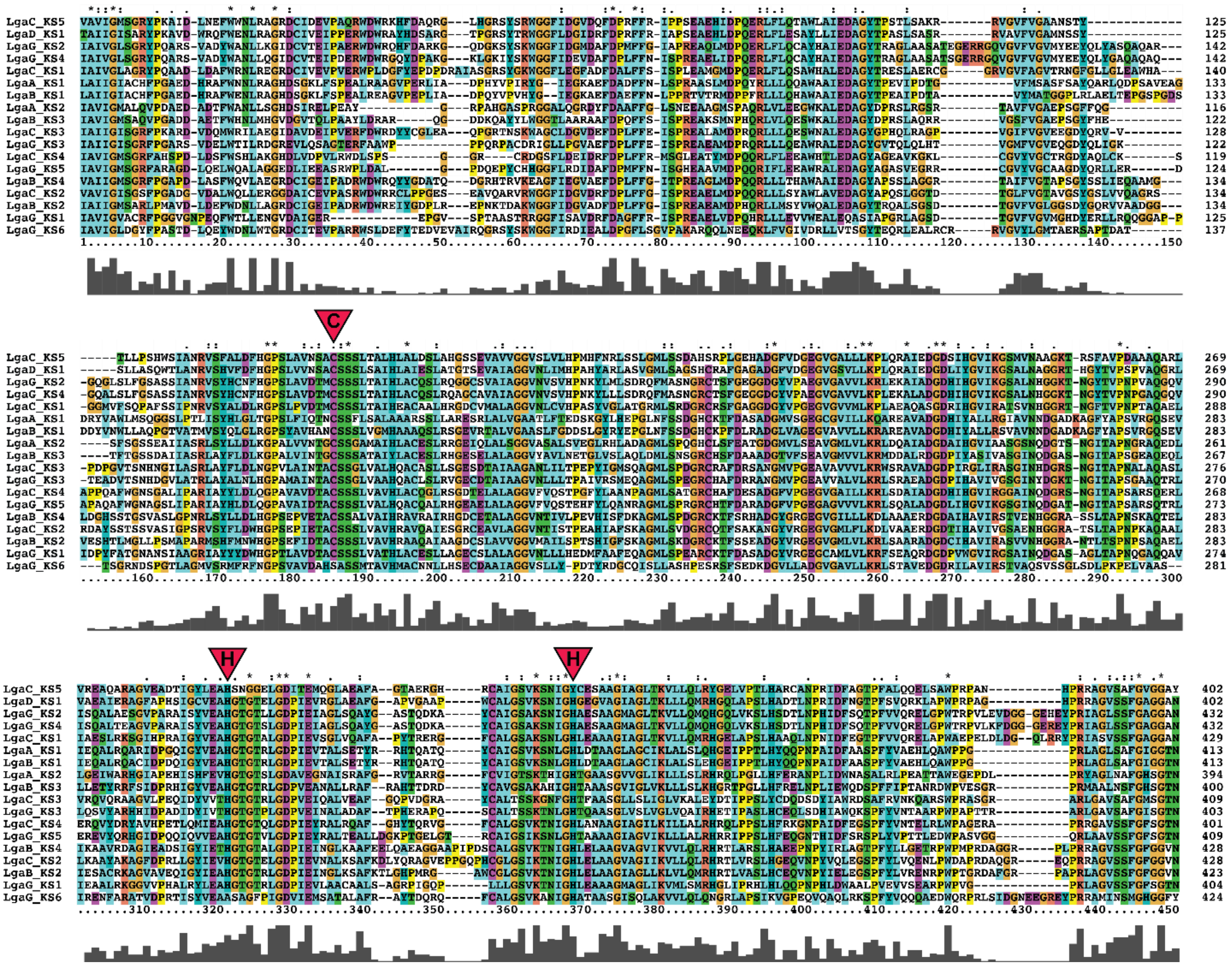

Supplementary Figure 16. Alignment of *lga* K domains. The catalytic triad (CHH) is highlighted.

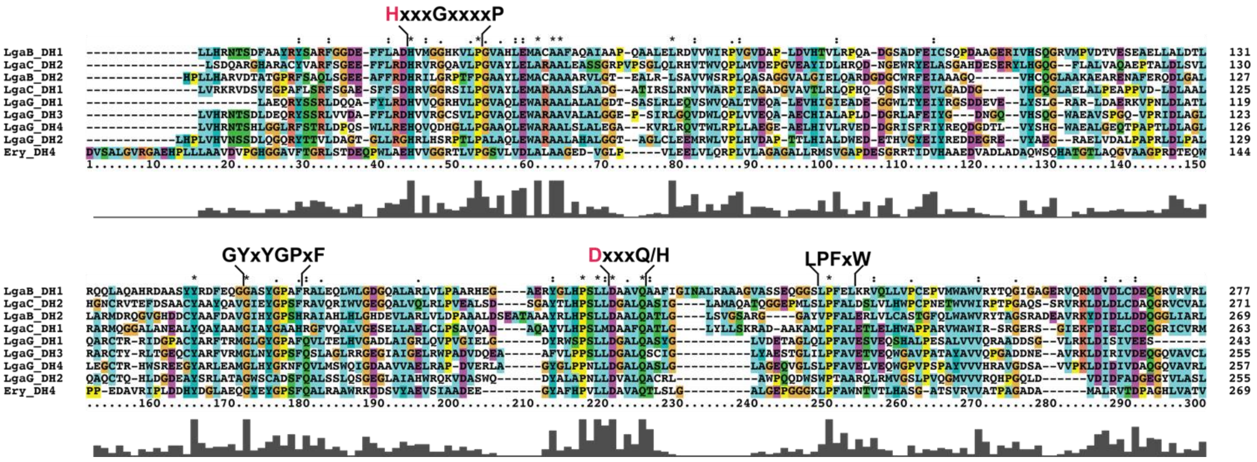

Supplementary Figure 17. Alignment of *lga* DH domains, along with one from the erythromycin biosynthetic gene cluster to allow comparison to previous alignments. The conserved HxxxGxxxxP, GYxYGPxF, DxxxQ/H and LPFxW motifs are highlighted. The catalytic H and D residues are shown in red.

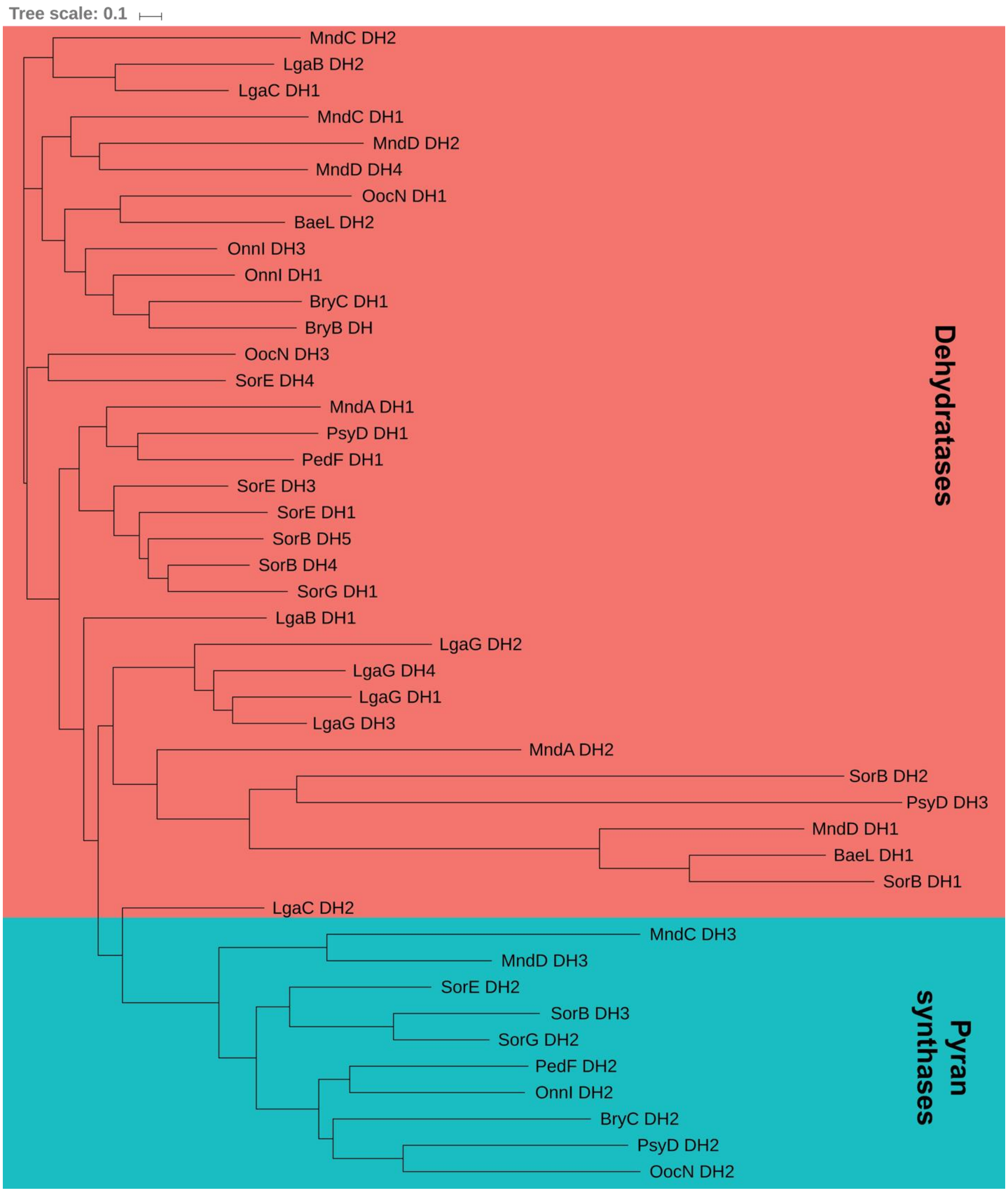

Supplementary Figure 18. Approximately maximum-likelihood tree based on DH and PS domains. The PS domains form a distinct clade as shown previously[1].

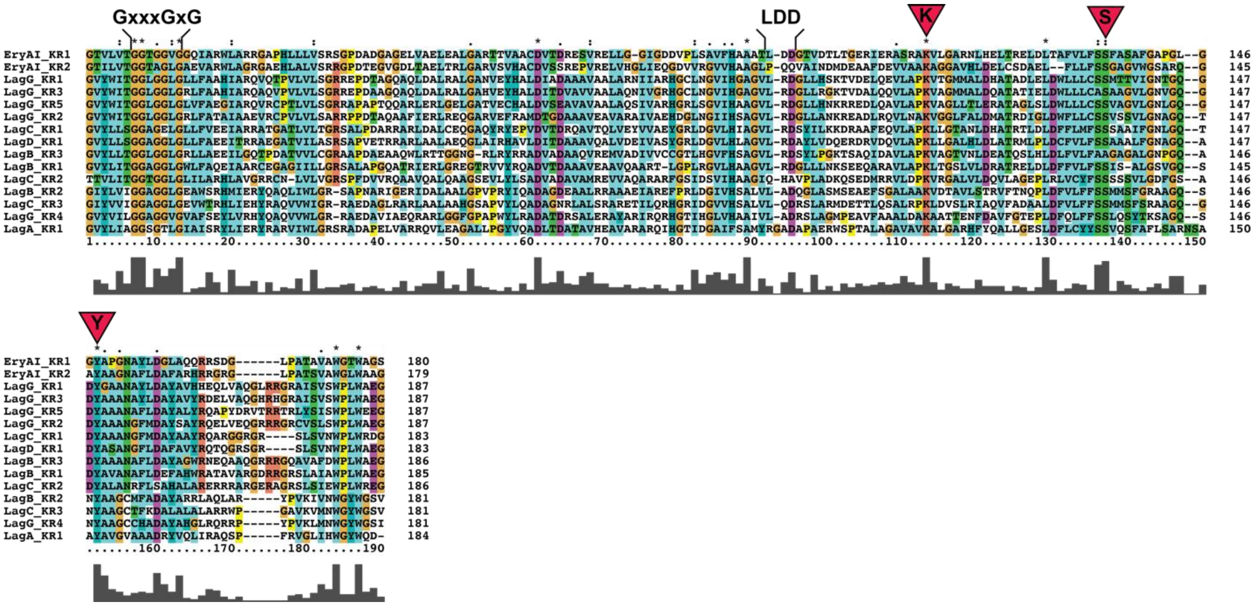

Supplementary Figure 19. Alignment of *lga* KR domains, along with two from the erythromycin biosynthetic gene cluster to allow comparison to previous alignments. The NADPH-binding site (GxxxGxG) is highlighted, as is the LDD motif that is thought to control product stereoconfiguration. The catalytic triad (KSY) is also highlighted.

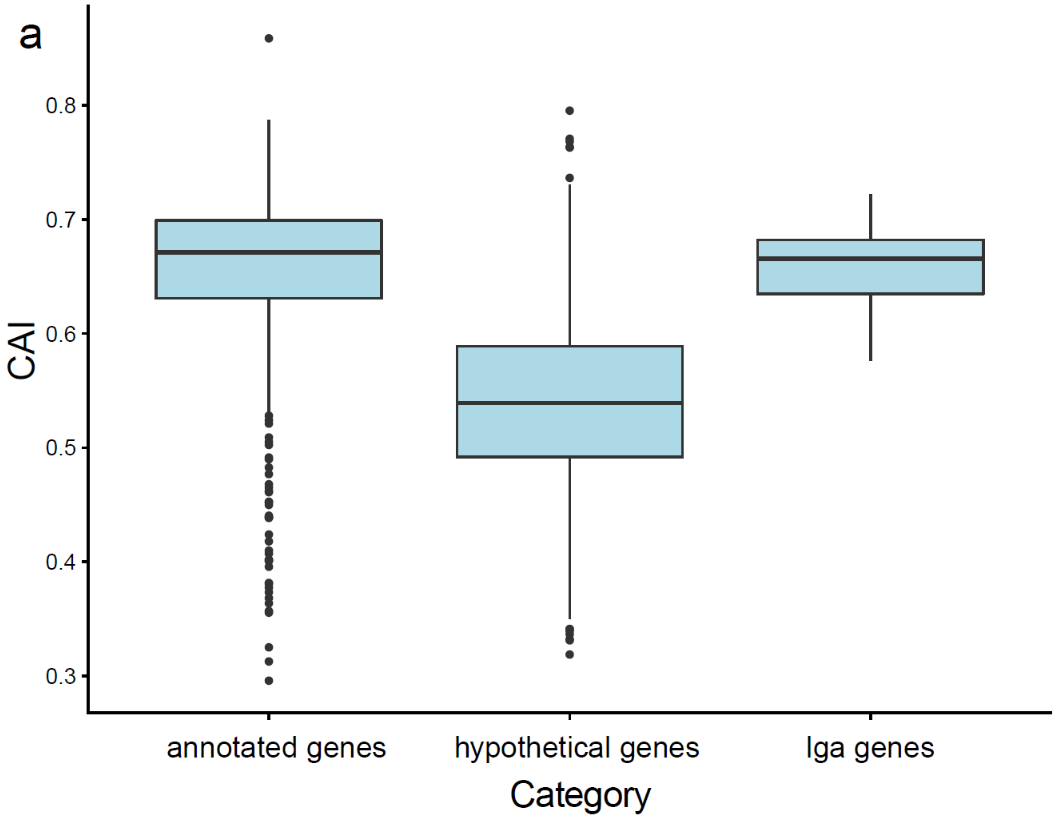

b

	annotated	hypothetical	lga
annotated		0.0000000	0.9865357
hypothetical	0.0000000		0.0000000
lga	0.9865357	0.0000000	

Supplementary Figure 20. Codon adaptation index (CAI) analysis comparing genes in the lagriamide biosynthetic cluster (*lga*) with all other genes in the *Burkholderia gladioli* Lv-StB genome. "Annotated genes" refer to any CDS not annotated as hypothetical. (a) Box plots representing the CAI of annotated, hypothetical and *lga* genes in the *B. gladioli* Lv-StB genome. The center value of the boxplot represents the median, the whiskers extend to the most extreme data point which is no more than 1.5 times the interquartile range, and outliers are represented by filled circles. (b) Individual p-values for pairwise comparisons of the groups compared through one-way ANOVA and Tukey HSD. Significant *P* values ($p < 0.05$) are highlighted in red.

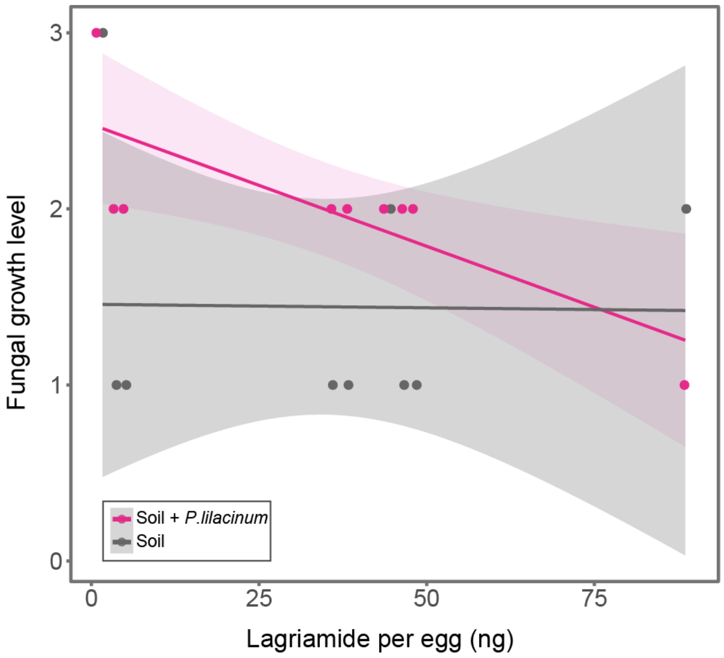

Supplementary Figure 21. Fungal growth levels on *Lagria villosa* eggs in relation to the amount of lagriamide detected in the same clutch. Data points in pink represent egg clusters exposed to field-collected soil inoculated with *Purpureocillium lilacinum* spores, showing a negative correlation between the two variables (Spearman Rank correlation, $p < 0.05$, rho = - 0.73, N = 9). Data points in dark gray represent egg clusters exposed to the same type of soil without additional treatment, showing no correlation between the variables (Spearman Rank correlation, $p > 0.05$, rho = -0.040, N = 9). Shadings represent 95% confidence intervals around the mean.

Supplementary Table 1. Characteristics of metagenomics bins obtained from the shotgun metagenome of *Lagria villosa.*

Cluster	Size (bp)	N50 (bp)	No. contigs	Coverage	Completeness (%)*	Purity (%)*	Taxonomic assignment†
DBSCAN_round1_3	2,126,292	8,170	301	1981	88.5	99.2	*B. gladioli* Lv-StB
DBSCAN_round4_2	3,579,730	83,015	56	336	66.9	98.2	*Comamonas* sp.
DBSCAN_round3_4	3,982,210	59,921	100	299	99.3	91.3	*Comamonas* sp.
DBSCAN_round25_31	56,809	4,677	12	214	23.0	90.6	*Ochrabactrum* sp. CDB2
DBSCAN_round316_1	1,035,061	6,469	162	198	30.9	97.7	*B. gladioli*
DBSCAN_round5_18	1,058,155	13,074	92	173	20.9	93.1	*B. gladioli*
DBSCAN_round3_6	4,650,003	27,531	252	171	84.9	100	*Herbaspirillum seropedicae*
DBSCAN_round5_2	6,999,405	30,038	341	109	60.4	91.7	*Variovorax paradoxus*
DBSCAN_round2_0	4,006,103	806,143	8	90	87.1	99.2	*Stenotrophomonas maltophilia*
DBSCAN_round5_13	3,199,337	12,058	321	84	33.8	100	*B. gladioli* Lv-StC
DBSCAN_round3_2	5,400,524	160,100	56	70	100	98.6	*Paraburkholderia* sp.
DBSCAN_round4_7	447,239	29,406	15	33	34.5	100	*Variovorax paradoxus*
DBSCAN_round58_1	3,626,665	18,913	248	32	27.3	94.7	*Pseudomonas lutescens*
DBSCAN_round434_184	17,401	17,401	1	24	20.9	100	*Pseudomonas* sp.
DBSCAN_round434_335	21,076	13.086	2	22.7	23.7	100	*Oxalobacter formigenes*
DBSCAN_round305_43	4,139,278	13,115	415	18	34.5	97.9	*Pseudomonas lutescens*
DBSCAN_round57_1	6,128,817	13,108	631	17	75.5	97.1	*Pseudomonas lutescens*
DBSCAN_round1_2	5,248,958	24,107	337	16	100	97.1	*Acinetobacter* sp.
DBSCAN_round4_0	8,660,577	260,679	66	8	91.4	96.1	*B. gladioli*
DBSCAN_round3_11	4,208,983	5,985	724	5	48.2	98.5	*Delftia acidovorans*

*Estimated based on the presence of single-copy marker genes

†Based on ANI or taxonomy assigned by Autometa.

Supplementary Table 2. Characteristics of single-cell genomes obtained from *Lagria villosa*.

Genome	Size (bp)	N50 (bp)	No. contigs	Completeness (%)*	Estimated genome size (Mbp)	*Lga* present?	Taxonomic assignment†
AG-701-A20	1,962,477	10,746	245	46.0	4.3	No	*Ochrobactrum pseudogrignonsense*
AG-701-C22	1,117,604	4,136	281	48.6	2.3	Yes	*B. gladioli* Lv-StB
AG-701-C23	1,055,127	10,158	138	19.6	5.4	No	*Acidivorax* sp.
AG-701-K20	504,320	4,590	118	15.5	3.3	No	*B. gladioli* Lv-StC
AG-701-O23	1,093,001	3,956	281	48.3	2.3	Yes	*B. gladioli* Lv-StB
AG-701-P21	593,052	7,509	94	13.8	4.3	No	*B. gladioli* Lv-StJ

*Estimated based on the presence of single-copy marker genes

†Based on the 16S rRNA gene sequence.

Supplementary references.

1. Pöplau, P., Frank, S., Morinaka, B. I. & Piel, J. An enzymatic domain for the formation of cyclic ethers in complex polyketides. *Angew. Chem - Int. Ed.* **52,** 13215–13218 (2013).
